# The association of white‐matter integrity with depression‐ symptoms among middle‐aged adults at high risk for Alzheimer's disease and related disorders

**DOI:** 10.1002/alz70857_105717

**Published:** 2025-12-26

**Authors:** Ramit Ravona‐Springer, Shiraz Vered, Abigail Livny, Ithamar Ganmore, Laili Soleimani, Tamar Shamir, Ganit Almog, Tal Niv, Shlomit Zorani, Adar Matatov, Joseph Azuri, Michal Schnaider Beeri, Galit Weinstein

**Affiliations:** ^1^ Geriatric psychiatry and memory unit, Sheba Medical Center, Ramat Gan, Israel; ^2^ Faculty of Medical and Health Science, Tel Aviv University, Tel Aviv, Israel; ^3^ The Joseph Sagol Neuroscience Center, Sheba Medical Center, Tel Hashomer, Israel; ^4^ School of Public Health, University of Haifa, Haifa, Mount Carmel, Israel; ^5^ The Sagol School of Neuroscience, Tel Aviv University, Tel Aviv, Israel; ^6^ Department of Diagnostic Imaging, Sheba Medical Center, Tel Hashomer, Israel; ^7^ Sheba Medical Center, Tel Hashomer, Israel; ^8^ Memory clinic, Sheba Medical Center, Tel Hashomer, Israel; ^9^ Icahn School of Medicine at Mount Sinai, New York, NY, USA; ^10^ The Joseph Sagol Neuroscience Center, Ramat Gan, Israel; ^11^ The Joseph Sagol Neuroscience Center, Sheba Medical Center, Ramat Gan, Israel; ^12^ The Joseph Sagol Neuroscience Center Sheba Tel‐HaShomer Medical Center, Ramat‐Gan, Israel, Israel; ^13^ Maccabi Health Services, Tel‐Aviv, Israel; ^14^ Herbert and Jacqueline Krieger Klein Alzheimer's Research Center, Piscataway, NJ, USA

## Abstract

**Background:**

Reduced white‐matter integrity, detectable before other brain imaging findings in individuals at risk for Alzheimer's disease and related disorders (ADRD), may underlie both the pathogenesis and consequences of depression. Investigating the relationship between white‐matter integrity and depressive symptoms in middle‐aged individuals at high risk for ADRD can provide insights into early markers of neurodegeneration.

**Method:**

We examined 301 cognitively normal offspring (mean age 54.6±6.9 years; 59.8% female) of ADRD patients from the Israel Registry for Alzheimer's Prevention (IRAP) study. Diffusion tensor imaging (DTI) was used to assess white matter integrity through fractional anisotropy (FA) and mean diffusivity (MD). Depressive symptoms were evaluated using the Center for Epidemiological Studies‐Depression (CESD) scale. Linear and logistic regression models, adjusting for age, sex, education, and the time between depression assessment and MRI acquisition, were used to analyze associations between white matter integrity and depressive symptoms.

**Result:**

The mean CESD score was 10.9±7.8 representing sub‐ syndromal depression. Higher FA in the genu and cingulum adjacent to the corpus callosum was associated with lower CESD scores (β=‐46.8; SE=16.5; *p* = 0.005 and β=‐68.2; SE=21.5; *p* = 0.002, respectively) and decreased odds of clinical depression (OR [95% CI]=0.89 [0.80‐0.99] and OR[95% CI]=0.86 [0.75‐0.98], respectively). No significant associations were found between FA in other white matter tracts or MD measures and depressive symptoms.

**Conclusion:**

In cognitively normal individuals at increased ADRD risk, disrupted white matter integrity in specific tracts is associated with more depressive symptoms already in midlife. These findings highlight the potential of white matter alterations as early biomarkers of neurodegeneration, creating opportunities for timely intervention and prevention.

